# The Uses of Costing

**Published:** 1919-09-06

**Authors:** 


### The Uses of Costing.

Under this title the Ministry of Reconstruction issues one of its weekly pamphlets at twopence, and urges the application of the practice to everyday life in the house, the garden, the farm, and the factory. "In its broad outline costing is the practice of measuring the use of materials and energy in all fields as a guide to action." The root idea is that it is as wicked to misuse a "labour hour" as it is to misuse a sovereign; that the manager of a factory has the same responsibility as to labour hours as a trustee has with regard to the moneys committed to his charge. An estimate of the labour expended to produce any given result is the first step towards economy of labour, and usually affords the indication as to how economies should be affected.

### Application to the Home.

The part of the pamphlet that will attract most attention is its application to the home, showing the waste of energy which the want of arrangement of many kitchens entails, and indicating lines along which improvements may be made. We note that these hinge largely upon the wheeled table. It is a type which was evolved, we believe, for use in the hospital theatre or ward. Of recent years we have seen it being more largely used in restaurants, first for cigars and cigarettes, then for liqueurs, more lately for pastries, hors d'œuvres and salads. Its use in the home for afternoon tea, or even for assistance in laying the table, would be an enormous saving of labour. It postulates the abolition of basements, of steps down and up such as are seen in old country houses, almost, it seems, of carpets in order that the table may be wheeled about.

### The Passing of the Basement House.

Of all devices of the mid-Victorian era we suppose the basement was the most prolific of waste of labour. In this age of increased production it stands condemned on this as much as it does on other accounts. Dark, damp, cold, and badly lighted, it has long been relegated to an hygienic standard which falls far short of the ideals of the present age. Morally it is as bad as it is economically and physically. It stigmatises the servants who live in almost as different beings from those who reside on the ground or first floors. The kitchens are shut off from the rest of the house as though they were parts of which it was necessary to be ashamed. Their position in the house was on a par with the word "menials," which described the tired and overworked women who inhabited them. Their absence in the well-planned house of to-day will be regretted only by the pseudo-aristocratic survivors of genteel poverty, to whom the merry laugh of the maid who serves them is as shocking as her short skirt or her desire for an evening at the pictures.

